# Predicting factors for breast cancer screening in Middle Eastern women based on health belief model: a systematic review

**DOI:** 10.1186/s43046-022-00150-3

**Published:** 2022-12-05

**Authors:** Narjes Bahri, Fariba Mardani, Neda Sharifi, Sareh Dashti

**Affiliations:** 1 Department of Midwifery, Faculty of Medicine, Social Determinants of Health Research Center, Gonabad University of Medical Sciences, Gonabad, Iran; 2 Department of Midwifery, Marvdasht Shahid Motahari Hospital, Shiraz University of Medical Sciences, Shiraz, Iran; 3 Department of Midwifery, Faculty of Nursing and Midwifery, Mashhad Medical Sciences, Islamic Azad University, Mashhad, Iran

**Keywords:** Breast cancer, Health belief model, Mammography, Breast self-exam

## Abstract

**Background:**

Breast cancer screening can reduce mortality and improve the quality of life in affected women. The present study aimed to determine the predictive factors of breast cancer screening in Iranian women based on the health belief model (HBM).

**Methods:**

This review was conducted by searching electronic databases of Google Scholar; electronic databases, including Scopus, PubMed/MEDLINE, Cochrane Library, Web of Science, ProQuest, Embase, and Google scholar Magiran; and SID with the English keywords of “breast cancer,” “mammography,” “health belief model,” and “breast self-exam” and the equivalent Persian keywords. The results were evaluated based on the health belief model (HBM) constructs. Articles were evaluated for quality and the findings were extracted and reviewed.

**Results:**

A total of 8 relevant articles were selected for review. Women’s awareness of breast cancer screening methods was moderate in two studies and poor in two other studies. Among the constructs of HBM, knowledge, perceived susceptibility, perceived severity, perceived benefits, and action plan were poor in the majority of the studies, while perceived barriers, cues to action, and self-efficacy were mainly good.

**Conclusion:**

Considering the observed weakness of many HBM constructs, it is recommended that special attention be given to all HBM constructs in implementing HBM-based education programs.

**Supplementary Information:**

The online version contains supplementary material available at 10.1186/s43046-022-00150-3.

## Background

Breast cancer comprises nearly one-third of female cancers and is the second cause of death due to cancer after lung cancer. The incidence of breast cancer has dramatically increased in the USA during the past decade [1, 2]. The incidence rate of breast cancer in the USA has increased by 3% from 2012 to 2016, but the mortality rate due to breast cancer has decreased in the same time period [3]. In contrast to developed countries, the mortality rate due to breast cancer has increased in developing countries including the Middle East [4]. Furthermore, the extensive use of screening, patient identification, and diagnosis of breast cancer has resulted in an increased rate of diagnosed cases of breast cancer [5]. Previous studies indicated that performing breast cancer screening improved longevity and quality of life in cancer patients and was also cost-effective [6, 7].

It was reported that the level of knowledge of women about breast cancer prevention is important in their participation in breast cancer screening and early treatment [8]. The health belief model (HBM) is one of the most utilized education models in health education and disease prevention [9]. HBM was first designed by Rosenstock et al. in the 1950s [10]. This model was specifically for designing prevention programs and behavior change in the short term [10]. HBM is the first model that assesses the concept of perceived barriers to performing health behaviors [11]. HBM also demonstrates the relationship between belief and personal understanding about the threats as well as the barriers and benefits of the health behavior [12]. Based on the HBM, preventive behavior requires the individuals to feel that they are in danger by the health problem (perceived susceptibility) and then understand the extent of the physical, mental, economic, and social effects of the health problem (perceived severity), receive positive signs from either internal or external environment (cues of action), and believe the benefits of the health behavior (perceived benefits) are more than the barriers to perform the health behavior (perceived barriers) and to achieve the behavior by judgment and competence (self-efficacy) [13–16].

A number of studies used HBM to assess the effective parameters and barriers in performing breast cancer screening behaviors [17–20]. The results of these studies have been controversial and each study focused on a specific construct of HBM. Furthermore, interventions have also been designed to improve breast cancer screening practices among women using the HBM [21–23]. Therefore, HBM can be a suitable model to assess the condition as well as the effectiveness of behavioral interventions in this regard [19, 24]. It seems that combining the findings of the previous studies can provide a framework for further clinical studies in this field by identifying the predictors for performing breast cancer screening. Therefore, the aim of this systematic review was to assess the predictors of performing breast cancer screening in Middle Eastern women in published studies that were conducted based on HBM.

## Methods

### Study design

This systematic review was conducted using the Preferred Reporting Items for Systematic Reviews and Meta-Analyses (PRISMA).

### Search strategy

The study was conducted by searching published articles in Persian and English languages in databases including PubMed, Scopus, SID, Magiran, and Google Scholar before November 2019. The search terms included “breast cancer,” “health belief model,” “mammography,” and “breast self-examination” and their Persian translations combined with Boolean operators. The search strategy was then designed based on Medical Subjects Heading (MeSH) terms (Additional file 1).

Inclusion criteria were original cross-sectional or intervention articles in Persian or English language that used HBM in the study design. Review articles, short communications, letter to editors, congress abstracts, studies that assessed screening for cancers other than breast cancer or used combined models, and studies with low quality were excluded from the review. The HBM constructs include risk susceptibility, risk severity, benefits to action, barriers to action, self-efficacy, and cues to action [25]. These constructs are mainly evaluated through specifically designed questionnaires [26].

**Fig. 1:**
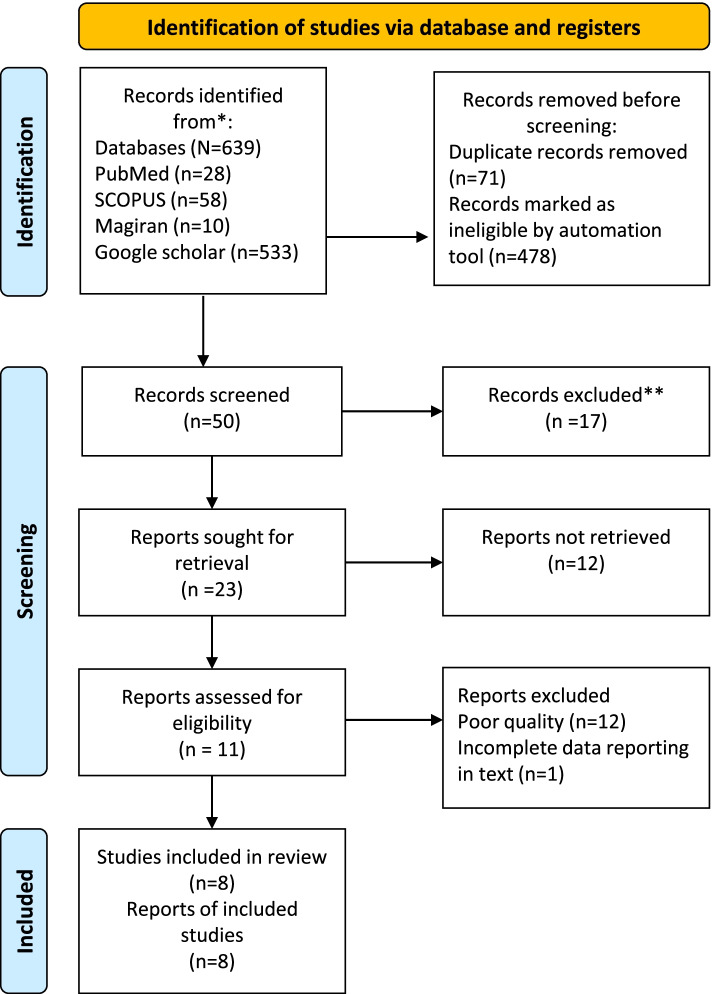
Successive steps in the selection of studies—PRISMA flow diagram

A database search was performed by one researcher. The retrieved studies were entered into Endnote® software and the duplicates were removed. Then, two researchers screened the studies independently based on title and abstract in order to reduce the risk or to minimize the risk of information, selection, and analysis biases. In case of disagreement between the researchers, a third researcher was invited to make the decision. Then, the full text of the included studies was obtained and the studies were reviewed by the two researchers independently and the risk of bias was evaluated. Then, the studies were screened for quality and studies with low quality were excluded. In case of disagreement between the reviewers, the opinion of a third researcher was asked. In the final step, the data in the remaining studies were extracted and presented in tables. Two authors independently assessed the methodological quality of each study using the JBI critical appraisal checklists. Disagreements were resolved by discussion with a third reviewer. The flowchart of the procedure of study selection is presented in Fig. 1.

### Risk of bias assessment for individual studies

The methodological quality of the included studies was evaluated based on the risk of bias assessment. The Joanna Briggs Institute critical appraisal checklists (JBI) were used to assess the risk of bias. The aims of the JBI critical appraisal tools are to assess the methodological quality of a study and to determine to what extent a study addressed the possibility of bias in its design, conduct, and data analysis [27]. The critical appraisal checklist for prevalence studies has 9 criteria that are scored based on a 2-point Likert scale, which indicates yes (1) and no (0). The JBI criteria appraisal checklist for prevalence studies includes appropriateness of sampling frame, appropriateness of sampling, adequacy of sample size, appropriateness of study settings, sufficient coverage of statistical analysis, validity of methods, reliability of measuring the outcome condition, appropriateness of statistical analysis, and adequacy of response rate. The JBI criteria appraisal checklist for randomized controlled trial studies includes 13 criteria: appropriateness of randomization, appropriateness of allocation concealment, matching at baseline, blinding of subjects and outcome assessors, similarity in receiving treatment other than intervention, follow-up, analysis, similarity of outcome measurement between intervention and control groups, reliability of outcome measures, appropriateness of statistical analysis, and appropriateness of the study design. Therefore, the JBI score for prevalence studies ranges between 0 and 9. Scores 1–3 were considered weak, scores 4–6 were considered moderate, and scores 7–9 were considered as good. In terms of intervention studies, scores 1–3, 4–7, 8–10, and 11–13 were considered weak, moderate, and good, respectively. Then, an overall appraisal was made to decide whether to include or exclude the studies [28]. Prevalence studies that acquired the minimum score of 3 and intervention studies that acquired the minimum score of 4 were considered to have acceptable quality and were included in the review.

### Quality assessment for individual studies

The JBI critical appraisal was also used for the quality assessment of the articles. Similar to risk of bias assessment, JBI critical appraisal items pertain to the methodological quality of the articles as per study design. Therefore, studies that were determined to be good based on the JBI critical appraisal were also considered to have acceptable methodological quality.

The PRISMA checklist of the study is presented in Additional file 2.

## Results

A total of 8 studies with overall 2897 participants were eligible for the review. Among the studies, only one study was conducted in Turkey. The rest of the studies were conducted in Iran. A summary of the findings of the studies is summarized in Table 1.

**Table 1:** Level of the reported constructs of HBM in the reviewed studies

Author (year)	Study characteristics			Health belief model constructs								
	Setting	*N*	Study population	Knowledge	Perceived susceptibility	Perceived severity	Perceived benefits	Perceived barriers	Cues to action	Action plan	Self-efficacy	Behavior
Taymoori (2014) [29]	Sanandaj, Iran	593	Clusters of women referring to health centers (>40 years old)	-	Poor	Poor	Poor	Poor	Poor	-	Poor	-
Mokhtari (2014) [30]	Khoy, Iran	162	Clusters of women referring to health centers (mean age 29 years old)	-	Moderate	-	Good	Good	Good	Moderate	-	Moderate
Heydari (2017) [31]	Bushehr, Iran	400	Teachers (>40 years old)	-	Poor	Poor	Poor	Poor	Poor	-	Poor	-
Jadgal (2016) [32]	Zahedan, Iran	240	Clusters of women referring to health centers (mean age 28 years old)	-	Good	Good	Good	Good	Good	-	-	Moderate
Hajian-Tilaki (2012) [33]	Babol, Iran	500	Clusters of women referring to health centers (18–65 years old)	Poor	Poor	Poor	Poor	Good	Good	Poor	Good	Poor
Karayurt (2007) [34]	Izmir, Turkey	430	Women referring to health centers (20–60 years old)	-	Good	Good	-	Good	Good	-	Good	-
Masoudi (2015) [35]	Dezful, Iran	226	Clusters of women referring to health centers (20–60 years old)	Poor	Poor	Poor	Poor	Poor	-	Poor	Poor	-
Shahraee (2013) [36]	Bushehr, Iran	400	Women referring to health centers (20–50 years old)	-	Moderate	Poor	Poor	Moderate	-	-	Moderate	poor

In the study by Taymoori et al. [29] on 593 women who referred to Sanandaj city health centers, north-west Iran, the constructs of HBM were assessed. The assessment was performed using a questionnaire, which consisted of perceived susceptibility (3 items), perceived severity (7 items), perceived benefits (6 items), perceived barriers (11 items), self-efficacy (10 items), and cue to action (7 items). Questionnaire items were scored based on a 5-point Likert scale. The scores in each construct were divided into poor, moderate, and good. The mean scores for all constructs were categorized as poor [29].

In the study by Mokhtari et al. [30], the correlation between health beliefs and breast cancer screening behaviors was assessed among women who referred to Khoy city health centers, north-west Iran. The study instrument was a 34-item questionnaire on HBM constructs, including perceived susceptibility, perceived severity, cue to action, perceived benefits, and perceived barriers. The total score for the questionnaire ranged from 34 to 170. Scores between 34 and 79 were considered weak, scores between 80 and 124 were considered moderate, and scores between 125 and 170 were considered strong belief. The mean total score of the subjects was 127.3 ± 17. Strong health beliefs were reported in 45.7% of the subjects, while 54% had moderate health beliefs. Perceived susceptibility was moderate among the subjects. Strong beliefs in perceived severity, cue to action, and perceived benefits and barriers of BSE were observed in 46.9%, 82.1%, 82.75, and 56.2% of the study subjects, respectively. Moderate beliefs regarding mammography were reported in 65% of the study subjects [30].

In the study by Heydari et al. (2017), the predictive factors for SBE behaviors among 20- to 50-year-old women were assessed based on HBM. The study was conducted on 400 women who referred to Bushehr health centers, south Iran. The study instrument was a questionnaire consisting of knowledge (24 items), perceived susceptibility (5 items), perceived severity (7 items), perceived benefits (6 items), perceived barriers (6 items), self-efficacy (11 items), cue to action (7 items), internal control (6 items), and external control (6 items), as well as chance control (6 items). The items were scored based on a 5-point Likert scale. Scores in each construct were divided into weak, moderate, and strong groups. The mean scores in perceived susceptibility, perceived severity, perceived benefits, perceived barriers, and self-efficacy were weak among women who did not perform BSE [31].

In the study by Jadgal et al. [32], the predictive factors for breast cancer preventive behaviors among junior high school teachers were assessed in Zahedan city, south-east Iran. The study was conducted on 240 female teachers using a knowledge questionnaire as well as an HBM construct questionnaire that consists of perceived susceptibility (6 items), perceived severity (5 items), perceived benefits (5 items), perceived barriers (5 items), self-efficacy (5 items), cue for action (6 items), and behavior (5 items). The items were scored based on a 5-point Likert scale. The total score was divided into weak, moderate, and good. The mean knowledge score of the study subjects was good. The mean scores for perceived susceptibility (22.56 ± 3.02), perceived severity (18.55 ± 3.57), perceived barriers (17.04 ± 3.63), self-efficacy (16.82 ± 3.28), and behavior (11.12 ± 2.59) were categorized as good [32].

In the study by Hajian-Tilaki et al. [33], the role of different health belief model components in practice of breast cancer screening was assessed among Iranian women. The study was conducted on 500 women aged 18–65 years who resided in an urban population in Babol city, North Iran. Study instruments were questionnaires regarding the practice of breast self-examination (BSE), breast clinical examination (BCE), and mammography as well as a standard health belief model questionnaire. Subjects who performed BSE and BCE had significantly higher mean scores in perceived benefit, self-efficacy, and health motivation, but no significant difference was reported between the scores of subjects who performed mammography and those who did not perform mammography. No significant difference was observed in perception of susceptibility, seriousness, and barriers between subjects with positive and negative behaviors. There was a significant positive association between scores of perceived benefits, perceived confidence/self-efficacy, and health motivation and performing BSE but not for mammography. No significant association was found between screening behaviors and scores of perceived susceptibility, perceived seriousness, and barriers. A strong association was observed between positive attitudes toward perceived benefits, perceived confidence/self-efficacy, and health motivation and performing BSE and BCE. The authors also indicated that the impact of HBM constructs on breast cancer screening may be influenced by culture and values [33].

In the study by Karayurt et al. [34] the Champion’s Revised HBM Scale for Turkish women was adapted to assess the association between selected sociodemographic variables and BSE. The study was conducted on 430 females who were living in one of the Health Center areas in Izmir, west Turkey. The study reported that subjects with low scores on barriers and those with high scores in confidence, perceived benefits, health motivation, perceived susceptibility, and perceived severity reported a high frequency of BSE practice in the last year. High school and university graduates, women with a family history of breast cancer, and women with breast cancer and those who received BSE training had a high frequency of SSE frequency in the past year [34].

In the study by Masoudi et al. [35], the predictors of breast cancer screening behavior of women who referred to health centers in Dezful, south Iran, were assessed based on HBM. The study showed that the knowledge and performance of the subjects were poor. There was a significant relationship between performance and knowledge, perceived susceptibility, perceived benefits, perceived barriers, self-efficacy, and cues to action. Predictors of performance were knowledge, perceived susceptibility, and self-efficacy [35].

In the study by Sahraee et al. [36], the predicting factors of BSE were assessed based on HBM and the locus of control model. The study was conducted on 400 women between the ages of 20 and 50 years old. The study instrument was the Champion’s Scale, health locus of control, and demographic and functional questionnaires. The study findings showed that regular BSE performance was reported in 10.9% of the subjects. Perceived self-efficacy was the strongest positive predictor of BSE performance (Exp (*B*) =1.863). Awareness had direct and indirect effects on the BSE [36].

The overall absolute and relative frequency distribution of the HBM constructs of the reviewed studies are presented in Table 1. None of the studies reported a good level of knowledge, while good levels of perceived susceptibility, perceived severity, perceived benefits, perceived barriers, self-efficacy, and cue to action were reported in 37.5%, 25.0%, 37.5%, 37.5%, 40.0%, and 66.6% of the articles, respectively.

## Discussion

The findings of this review showed that the perceived susceptibility score of the studied women about breast cancer screening was either poor or good and no case of moderate scores was reported. Similar findings were reported in a previous study regarding the perceived susceptibility scores in terms of cervical cancer preventive behaviors [37]. In contrast, Kasmaei et al. reported that the scores of perceived susceptibilities regarding cervical cancer screening behavior were good [38]. In a study on American college students, perceived susceptibility was low in all 342 participants [39].

The findings of this review also showed that the scores of perceived severity about breast cancer screening methods were poor in the majority of cases but ranged from poor to good. Similarly, in a study on nutritional practices of pregnant women, the scores of perceived severity were reported to be low in the majority of Iranian pregnant subjects [40]. In contrast, in other studies on Iranian subjects regarding preventive behavior about brucellosis in the general population and anxiety preventive behavior in pregnant women, the score of perceived severity was reported to be good and moderate among study subjects, respectively [41, 42]. In a study on 342 American college students, the mean perceived severity score was 14.4 from 28, which was considered as low to moderate, while in another study on 1967 women in Indonesia, the mean perceived severity score was high (38.63) [39, 43]. These findings indicate that the level of perceived severity differs based on population, region, culture, and subject.

The findings of this review showed that the scores of perceived benefits were low in the majority of subjects but ranged from poor to good. Similarly, in a study on Ethiopian women, only 27.5% of the participants believed in benefits of breast self-examination [44]. In a study conducted in Indonesia, the mean score for perceived benefits of breast cancer screening was moderate [20]. These findings indicate that similar to other constructs of HBM, perceived benefits of breast cancer screening are affected by geographical characteristics of the participants. In previous studies on nutritional behaviors of pregnant women, behavior towards physical maturation in female students, and preventive behavior for failure to thrive, the scores of perceived benefits were good [45–47].

The findings of this review also showed that the scores of perceived barriers were either poor or good in the studied subjects. None of the studies reported moderate perceived barriers in our review. This finding was in line with the findings of previous studies in Taiwan and Indonesia that reported poor level of perceived barriers regarding breast cancer screening behaviors [20, 48]. In a previous study, the level of perceived barriers regarding decision-making on the method of delivery in Iranian primiparous women was reported to be moderate [49]. In another study on hypertension management behaviors in the Iranian elderly, the level of perceived barriers was reported to be good [50].

The findings of this review showed that the self-efficacy scores of the studied subjects were either poor or good but the majority of the subjects had poor levels of self-efficacy. In previous studies on breast cancer screening behaviors in Malta and Ethiopia, self-efficacy was reported to be good [51, 52]. In previous studies on Iranian subjects regarding hypertension management behaviors in the elderly and cervical cancer screening behaviors, the level of self-efficacy was moderate [50, 53]. As mentioned before, the reason for the different findings between the studies might be due to the differences in regional, cultural, and the investigated subjects as well as differences in study designs and sample sizes.

The findings of this review also showed that the cues to action scores of the studied subjects were either poor or good. In previous studies on nutritional behaviors in Iranian pregnant women, the scores for cues to action were reported to range between moderate and good [40, 46].

Although not a distinct construct of HBM, knowledge is believed to be indirectly related to HBM constructs. The findings of this review showed that the overall knowledge of the women regarding breast cancer screening was low to moderate. None of the studies reported an adequate or good level of knowledge about breast cancer screening. This finding was in line with the findings of previous studies that assessed different constructs of HBM. In a previous study in 2012, the level of knowledge of Iranian women regarding cervical cancer preventive behaviors was reported to be moderate in the majority of subjects [54]. In a narrative review conducted on knowledge, beliefs, and attitudes towards breast cancer screening in Latin America, the percentage of women with some knowledge about breast screening ranged from 50% in Trinidad to 90% in Brazil [55]. However, the mentioned review in Latin America did not assess knowledge based on HBM. Similarly, in another study on cervical cancer preventive behaviors, the majority of the subjects were reported to have a moderate level of knowledge [53]. Only in one Iranian study conducted by Tahmasebi et al., the level of knowledge regarding cervical cancer screening was reported to be good [37]. A reason for the observed difference between the findings of this study and the previous studies might be related to the study design. The study by Tahmasebi et al. was a clinical trial (50 subjects in the intervention group and 50 subjects in the control group) that cannot reliably assess the prevalence of different levels of knowledge.

Overall, the findings of this review showed that Middle Eastern women had a poor status in knowledge, perceived susceptibility, and perceived severity, while their status in other HBM constructs ranged from poor to good. Considering the HBM theory, lack of knowledge is related to poor understanding about the other constructs, including perceived susceptibility, perceived severity, and perceived barriers. These constructs, in part, affect self-efficacy and cue to action. Based on these observations and considering the HBM theory, it can be deduced that in studies that reported a low level of knowledge in terms of breast cancer screening, the level of other HBM constructs should be low. This theory was proven in the reviewed studies. Previous studies have also indicated that when the level of knowledge of participants was low, their health behavior was also low and the interventions that affected the level of knowledge of participants regarding health behavior were improved [56–58]. However, the findings of previous studies about the effect of perceived severity and susceptibility and health behavior were controversial. Some studies showed that the relationship between perceived severity and susceptibility was not necessarily related to healthy behavior [59, 60], while other studies indicated that these constructs were related to health behavior [61, 62]. The findings of the current review indicated that these constructs were related to breast cancer screening behavior. Therefore, it can be concluded that the HBM theory can be used to determine the breast cancer screening behavior of Middle Eastern women. In order to improve the breast cancer screening behavior of Middle Eastern women, interventions should focus on knowledge and empowerment of the at-risk population based on HBM theory.

To the best of our knowledge, this systematic review was the first study that tried to synthesize the results of previous studies about predicting factors for breast cancer screening in Middle Eastern women. One of the limitations of this review was including studies that used different questionnaires for assessing the constructs of HBM regarding breast cancer prevention behavior. We suggest that the future studies focus on developing standard questionnaires regarding HBM constructs for preventing behaviors of breast cancer.

## Conclusion

Considering the findings of this review, HBM can be used to predict breast cancer screening behavior of Middle Eastern women. This theory can be used to improve breast cancer screening behavior. Interventions should consider all HBM constructs as the studied women had poor status in the majority of these constructs. Regarding the crucial role of knowledge, it is recommended that education programs should be designed to improve all constructs of the HBM model regarding breast cancer screening.

## Supplementary Information

**Additional file 1.** Search strategy for the systematic review.

**Additional file 2.** PRISMA checklist of the review.

## Data Availability

Not applicable.

## Abbreviations

BCE: Breast clinical examination
BSE: Breast self-examination
HBM: Health belief model
JBI: Joanna Briggs Institute
MeSH: Medical Subjects Heading
PRISMA: Preferred Reporting Items for Systematic Reviews and Meta-Analyses
