# Supplementary material for: Predicting factors for breast cancer screening in Middle Eastern women based on health belief model: a systematic review
Source: J Egypt Natl Canc Inst. 2022 Dec 5;34:50. doi: 10.1186/s43046-022-00150-3 (PMC13314224; doi:10.1186/s43046-022-00150-3)
Supplement: Supplementary file 1 — Additional file 1. Search strategy for the systematic review. [file 43046_2022_150_MOESM1_ESM.docx]

**Predicting Factors for Breast Cancer Screening in Middle-Eastern Women Based on Health Belief Model: A Systematic Review**

**Abstract**

**Background:** Breast cancer screening can reduce mortality and improve quality of life in affected women. The present study aimed to determine the predictive factors of breast cancer screening in Iranian women based on the Health Belief Model (HBM).

**Methods:** This review was conducted by searching electronic databases of Google Scholar, electronic databases, including Scopus, PubMed/Medline, Cochran library, Web of Science, ProQuest, Embase, and Google scholar Magiran, and SID with the English keywords of "breast cancer", "mammography", "health belief model", "breast self-exam", and the equivalent Persian keywords. The results were evaluated based on the health belief model (HBM) constructs. Articles were evaluated for quality and the findings were extracted and reviewed.

**Results:** A total of 8 relevant articles were selected for review. Women's awareness of breast cancer screening methods was moderate in two studies and poor in two other studies. Among the constructs of HBM, knowledge, perceived susceptibility, perceived severity, perceived benefits, and action plan were poor in majority of the studies, while perceived barriers, cues to action, and self -efficacy were mainly good.

**Conclusion:** Considering the observed weakness many HBM constructs, it is recommended that special attention be given to all HBM constructs in implementing HBM-based education programs.

**Keywords:** Breast Cancer, Health Belief Model, Mammography, Breast Self-Exam

**Background**

Breast cancer comprises nearly one third of female cancers and is the second cause of death due to cancer after lung cancer. The incidence of breast cancer has dramatically increased in the United States during the past decade ([1](#_ENREF_1), [2](#_ENREF_2)). The incidence rate of breast cancer in the United States has increased by 3% from 2012 to 2016 but the mortality rate due to breast cancer has decreased in the same time period ([3](#_ENREF_3)). In contrast to developed countries, the mortality rate due to breast cancer has increased in developing countries including the Middle-East ([4](#_ENREF_4)). Furthermore, the extensive use of screening, patient identification and diagnosis of breast cancer has resulted in an increased rate of diagnosed cases of breast cancer ([5](#_ENREF_5)). Previous studies indicated that performing breast cancer screening improved longevity and quality of life in cancer patients and was also cost effective ([6](#_ENREF_6), [7](#_ENREF_7)).

It was reported that the level of knowledge of women about breast cancer prevention is important in their participation in breast cancer screening and early treatment ([8](#_ENREF_8)). Health belief model (HBM) is one of the most utilized education models in health education and disease prevention ([9](#_ENREF_9)). HBM was first designed by Rosenstock et al. in 1950s ([10](#_ENREF_10)). This model was specifically for designing prevention programs and behavior change in short term ([10](#_ENREF_10)). HBM is the first model that assesses the concept of perceived barriers to performing health behaviors ([11](#_ENREF_11)). HBM also demonstrates the relationship between belief and personal understanding about the threats as well as the barriers and benefits of the health behavior ([12](#_ENREF_12)). Based on the HBM preventive behavior requires the individuals to feel that they are in danger by the health problem (perceived susceptibility), and then understand the extent of the physical, mental, economic and social effects of the health problem (perceived severity), receive positive signs from either internal or external environment (cues of action), believe the benefits of the health behavior (perceived benefits), are more than the barriers to perform the health behavior (perceived barriers) and to achieve the behavior by judgment and competence (self-efficacy) ([13-16](#_ENREF_13)).

A number of studies used HBM to assess the effective parameters and barriers in performing breast cancer screening behaviors ([17-20](#_ENREF_17)). The results of these studies have been controversial and each study focused on a specific construct of HBM. Furthermore, interventions have also been designed to improve breast cancer screening practices among women using the HBM ([21-23](#_ENREF_21)). Therefore, HBM can be a suitable model to assess the condition as well as effectiveness of behavioral interventions in this regard ([19](#_ENREF_19), [24](#_ENREF_24)). It seems that combining the findings of the previous studies can provide a framework for further clinical studies in this field by identifying the predictors for performing breast cancer screening. Therefore, the aim of this systematic review was to assess the predictors of performing breast cancer screening in Middle-Eastern women in published studies that were conducted based on HBM.

**Methods**

***Study design***

This systematic review was conducted using the Preferred Reporting Items for Systematic Reviews and Meta-Analyses (PRISMA).

***Search strategy***

The study was conducted by searching published articles in Persian and English languages in databases including PubMed, Scopus, SID, Magiran and Google Scholar before November 2019. The search terms included “breast cancer”, health belief model”, “mammography”, and “breast self-examination” and their Persian translations combined with Boolean operators. Search strategy was then designed based on Medical Subjects heading (MeSH) terms (Supplementary file 1).

Inclusion criteria were original cross-sectional or intervention articles in Persian or English language that used HBM in study design. Review articles, short communications, letter to editors, congress abstracts, studies that assessed screening for cancers other than breast cancer or used combined models, and studies with low quality were excluded from the review. The HBM constructs include risk susceptibility, risk severity, benefits to action, barriers to action, self-efficacy, and cues to action ([25](#_ENREF_25)). These constructs are mainly evaluated through specifically designed questionnaires ([26](#_ENREF_26)).

Databases search was performed by one researcher. The retrieved studies were entered into Endnote® software and the duplicates were removed. Then two researchers screened the studies independently based on title and abstract in order to reduce the risk of to minimize the risk of information, selection, and analysis biases. In case of disagreement between the researchers, a third researcher was invited to make the decision. Then the full text of the included studies was obtained and the studies were reviewed by the two researches independently and the risk of bias was evaluated. Then the studies were screened for quality and studies with low quality were excluded. In case of disagreement between the reviewers, the opinion of a third researcher was asked. In the final step the data in the remaining studies were extracted and presented in tables. Two authors independently assessed the methodological quality of each study using the JBI critical appraisal checklists. Disagreements were resolved by discussion with a third reviewer. The flowchart of the procedure of study selection is presented in Figure 1.

***Risk of Bias Assessment for Individual Studies***

The methodological quality of the included studies was evaluated based on risk of bias assessment. The Joanna Briggs Institute critical appraisal checklists (JBI) were used to assess the risk of bias. The aims of the JBI critical appraisal tools are to assess the methodological quality of a study and to determine to what extent a study addressed the possibility of bias in its design, conduct and data analysis ([27](#_ENREF_27)) . The critical appraisal checklist for prevalence studies has 9 criteria that are scored based on a two-point Likert scale, which indicate yes (1) and no (0). The JBI criteria appraisal checklist for prevalence studies includes appropriateness of sampling frame, appropriateness of sampling, adequacy of sample size, appropriateness of study settings, sufficient coverage of statistical analysis, validity of methods, reliability of measuring the outcome condition, appropriateness of statistical analysis, and adequacy of response rate. The JBI criteria appraisal checklist for randomized controlled trial studies includes 13 criteria, appropriateness of randomization, appropriateness of allocation concealment, matching at baseline, blinding of subjects and outcome assessors; similarity in receiving treatment other than intervention, follow up, analysis, similarity of outcome measurement between intervention and control group, reliability of outcome measures, appropriateness of statistical analysis, appropriateness of the study design. Therefore, the JBI score for prevalence studies ranges between 0 and 9. Scores 1-3 were considered week, scores 4-6 were considered moderate, and scores 7-9 were considered as good. In terms of intervention studies scores 1-3, 4-7, 8-10, and 11-13 were considered week, moderate and good, respectively. Then an overall appraisal was made to decide whether to included or excluded the studies ([28](#_ENREF_28)). Prevalence studies that acquired the minimum score of 3 and intervention studies that acquired the minimum score of 4 were considered to have acceptable quality and were included in the review.

***Quality assessment for individual studies***

The JBI critical appraisal was also used for the quality assessment of the articles. Similar to risk of bias assessment, JBI critical appraisal items pertain to the methodological quality of the articles as per study design. Therefore, studies that were determined to be good based on the JBI critical appraisal were also considered to have acceptable methodological quality.

The PRISMA checklist of the study is presented in Supplementary files-2.

**Results**

A total of 8 studies with overall 2897 participants were eligible for the review. Among the studies only one study was conducted in turkey. The rest of the studies were conducted in Iran. Summary of the findings of the studies are summarized in Table 1.

In the study by Taymoori et al. (2014) on 593 women who referred to Sanandaj city health centers, north-west Iran, the constructs of HBM were assessed. Assessment was performed using a questionnaire, which consisted of perceived susceptibility (3 items), perceived severity (7 items), perceived benefits (6 items), perceived barriers (11 items), self-efficacy (10 items), and cue to action (7 items). Questionnaire items were scored based on a 5-point Likert scale. The scores in each construct was divided into poor, moderate and good. The mean scores for all constructs were categorized as poor ([29](#_ENREF_29)).

In the study by Mokhtari et al. (2014), the correlation between health beliefs and breast cancer screening behaviors was assessed among women who referred to Khoy city health centers, north-west Iran. The study instrument was a 34-items questionnaire on HBM constructs, including perceived susceptibility, perceived severity, and cue to action, perceived benefits, as well as perceived barriers. The total score for the questionnaire ranged from 34 to 170. Scores between 34 and 79 were considered week, scores between 80 and 124 were considered moderate and scores between 125 and 170 were considered strong belief. The mean total score of the subjects was 127.3 ± 17. Strong health beliefs was reported in 45.7% of the subjects, while 54% had moderate health beliefs. Perceived susceptibility was moderate among the subjects. Strong beliefs in perceived severity, cue to action, perceived benefits and barriers of BSE were observed in 46.9%, 82.1%, 82.75, and 56.2% of the study subjects, respectively. Moderate beliefs regarding mammography was reported in 65% of the study subjects ([30](#_ENREF_30)).

In the study by Hedayat et al. (2017), the predictive factors for SBE behaviors among 20- to 50-years-old women was assessed based on HBM. The study was conducted on 400 women who referred to Bushehr health centers, south Iran. The study instrument was a questionnaire consisting of knowledge (24 items), perceived susceptibility (5 items), perceived severity (7 items), perceived benefits (6 items), perceived barriers (6 items), self-efficacy (11 items), cue to action (7 items), internal control (6 items) and external control (6 items), as well as chance control (6 items). The items were scored based on a 5-point Likert scale. Scores in each construct was divided into week, moderate and strong groups. The mean scores in perceived susceptibility, perceived severity, perceived benefits, perceived barriers and self-efficacy were week among women who did not perform BSE ([31](#_ENREF_31)).

In the study by Jadgal et al. (2016), the predictive factors for breast cancer preventive behaviors among junior high school teachers were assessed in Zahedan city, south-east Iran. The study was conducted on 240 female teachers using a knowledge questionnaire as well as HBM construct questionnaire that consists of perceived susceptibility (6 items), perceived severity (5 items), perceived benefits (5 items), perceived barriers (5 items), self-efficacy (5 items), cue for action (6 items), and behavior (5 items). The items were scored based on a 5-point Likert scale. The total score was divided into week, moderate and good. The mean knowledge score of the study subjects was good. The mean scores for perceived susceptibility (22.56 ± 3.02), perceived severity (18.55 ± 3.57), perceived barriers (17.04 ± 3.63), self-efficacy (16.82 ± 3.28), and behavior (11.12 ± 2.59) were categorized as good ([32](#_ENREF_32)).

In the study by Hajian-Tilaki et al. (2014), the role of different health belief model components in practice of breast cancer screening was assessed among Iranian women. The study was conducted on 500 women aged 18-65 years who resided in an urban population in Babol city, north Iran. Study instruments were questionnaires regarding the practice of breast self-examination (BSE), breast clinical examination (BCE), and mammography as well as a standard health belief model questionnaire. Subjects who performed BSE and BCE had significantly higher mean scores in perceived benefit, self-efficacy, and health motivation, but no significant difference was reported between the scores of subjects who performed mammography and those who did not perform mammography. No significant difference was observed in perception of susceptibility, seriousness, and barriers between subjects with positive and negative behaviors. There was a significant positive association between scores of perceived benefits, perceived confidence/self-efficacy, and health motivation and performing BSE but not for mammography. No significant association was found between screening behaviors and scores of perceived susceptibility, perceived seriousness, and barriers. A strong association was observed between positive attitudes toward perceived benefits, perceived confidence/self-efficacy, and health motivation and performing BSE and BCE. The authors also indicated that the impact of HBM constructs on breast cancer screening may be influenced by culture and values ([33](#_ENREF_33)).

In the study by Karayurt et al. (2007) the Champion's Revised HBM Scale for Turkish women was adapted to assess the association between selected sociodemographic variables and BSE. The study was conducted on 430 females who were living in one of the Health Center areas in Izmir, west Turkey. The study reported that subjects with low scores on barriers and those with high scores in confidence, perceived benefits, health motivation, perceived susceptibility, and perceived severity reported a high frequency of BSE practice in the last year. High school and university graduates, women with a family history of breast cancer, and women with breast cancer and those who received BSE training had high frequency of SSE frequency in the past year ([34](#_ENREF_34)).

In the study by Masoudiyekta et al. (2015), the predictors of breast cancer screening behavior of women who referred to health centers in Dezful, south Iran were assessed based on HBM. The study showed that the knowledge and performance of the subjects were poor. There was a significant relationship between performance and knowledge, perceived susceptibility, perceived benefits, perceived barriers, self-efficacy, and cues to action. Predictors of performance were knowledge, perceived susceptibility, and self-efficacy ([35](#_ENREF_35)).

In the study by Sahraee et al. (20013), the predicting factors of BSE were assessed based on HBM and the locus of control model. The study was conducted on 400 women between the ages of 20 and 50 years old. The study instrument was the Champion’s Scale, health locus of control, and demographic and functional questionnaires. The study findings showed that regular BSE performance was reported in 10.9% of the subjects. Perceived self-efficacy was the strongest positive predictor of BSE performance (Exp (B) =1.863). Awareness had direct and indirect effects on the BSE ([36](#_ENREF_36)).

The overall absolute and relative frequency distribution of the HBM constructs of the reviewed studies are presented in Table 1. None of the studies reported good level of knowledge, while good level of perceived susceptibility, perceived severity, perceived benefits, perceived barriers, self-efficacy and cue to action were reported in 37.5%, 25.0%, 37.5%, 37.5%, 40.0% and 66.6%, of the articles respectively.

**Discussion**

The findings of this review showed that perceived susceptibility score of the studied women about breast cancer screening was either poor or good and no case of moderate scores was reported. Similar findings were reported in a previous study regarding the perceived susceptibility scores in terms of cervical cancer preventive behaviors ([37](#_ENREF_37)). In contrast, Kasmaei et al. (2014) reported that the scores of perceived susceptibilities regarding cervical cancer screening behavior were good ([38](#_ENREF_38)). In a study on American college students perceived susceptibility was low in all 342 participants ([39](#_ENREF_39)).

The findings of this review also showed that the scores of perceived severity about breast cancer screening methods were poor in majority of cases but ranged from poor to good. Similarly, in a study on nutritional practices of pregnant women, the scores of perceived severity was reported to be low in majority of Iranian pregnant subjects ([40](#_ENREF_40)). In contrast in other studies on Iranian subjects regarding preventive behavior about brucellosis in general population and anxiety preventive behavior in pregnant women, the score of perceived severity were reported to be good and moderate among study subjects, respectively ([41](#_ENREF_41), [42](#_ENREF_42)). In a study on 342 American college students the mean perceived severity score was 14.4 from 28, which was considered as low to moderate, while in another study on 1967 women in Indonesia, the mean perceived severity score was high (38.63) ([39](#_ENREF_39), [43](#_ENREF_43)). These findings indicate that the level of perceived severity differs based on population, region, culture and subject.

The findings of this review showed that the scores of perceived benefits were low in majority of subjects but ranged from poor to good. Similarly, in a study on Ethiopian women, only 27.5% of the participants believed in benefits of breast self-examination ([44](#_ENREF_44)). In a study conducted in Indonesia, the mean score for perceived benefits of breast cancer screening was moderate ([20](#_ENREF_20)). These findings indicate that similar to other constructs of HBM, perceived benefits of breast cancer screening is affected by geographical characteristics of the participants. Previous studies on nutritional behaviors of pregnant women, behavior towards physical maturation in female students, preventive behavior for failure to thrive, the scores of perceived benefits were good ([45-47](#_ENREF_45)).

The findings of this review also showed that the scores of perceived barriers were either poor or good in the studied subjects. None of the studies reported moderate perceived barriers in our review. This finding was in line with the findings of previous studies in Taiwan and Indonesia that reported poor level of perceived barriers regarding breast cancer screening behaviors ([20](#_ENREF_20), [48](#_ENREF_48)). In a previous study the level of perceived barriers regarding decision making on the method of delivery in Iranian primiparous women was reported to be moderate ([49](#_ENREF_49)). In another study on hypertension management behaviors in Iranian elderly, the level of perceived barriers was reported to be good ([50](#_ENREF_50)).

The findings of this review showed that the self-efficacy scores of the studied subjects were either poor or good but majority of the subjects had poor levels of self-efficacy. In previous studies on breast cancer screening behaviors in Malta and Ethiopia, self-efficacy was reported to be good ([51](#_ENREF_51), [52](#_ENREF_52)). In previous studies on Iranian subjects regarding hypertension management behaviors in the elderly and cervical cancer screening behaviors, the level of self-efficacy was moderate ([50](#_ENREF_50), [53](#_ENREF_53)). As mentioned before, the reason for the different findings between the studies might be due to the differences in regional, cultural and the investigated subjects as well as differences in study designs and sample sizes.

The findings of this review also showed that the cues to action scores of the studied subjects were either poor or good. In previous studies on nutritional behaviors in Iranian pregnant women, the scores for cues to action were reported to range between moderate and good ([40](#_ENREF_40), [46](#_ENREF_46)).

Although not a distinct construct of HBM, knowledge is believed to be indirectly related to HBM constructs. The findings of this review showed that the overall knowledge of the women regarding breast cancer screening was low to moderate. None of the studies reported adequate or good level of knowledge about breast cancer screening. This finding was in line with the findings of previous studies that assessed different constructs of HBM. In a previous study in 2012, the level of knowledge of Iranian women regarding cervical cancer preventive behaviors was reported to be moderate in majority of subjects ([54](#_ENREF_54)). In a narrative review conducted on knowledge, beliefs and attitudes towards breast cancer screening in Latin America, percentage of women with some knowledge about breast screening ranged from 50% in Trinidad to 90% in Brazil ([55](#_ENREF_55)). However, the mentioned review in Latin America did not assess knowledge based on HBM. Similarly, in another study on cervical cancer preventive behaviors, majority of the subjects were reported to have moderate level of knowledge ([53](#_ENREF_53)). Only in one Iranian study conducted by Tahmasebi et al. (2016), the level of knowledge regarding cervical cancer screening was reported to be good ([37](#_ENREF_37)). A reason for the observed difference between the findings of this study and the previous studies might be related to the study design. The study by Tahmasebi was a clinical trial (50 subjects in intervention group and 50 subjects in the control group that cannot reliably assess the prevalence of different levels of knowledge.

Overall the findings of this review showed that the Middle Eastern women had a poor status in knowledge, perceived susceptibility, and perceived severity, while their status in other HBM constructs ranged from poor to good. Considering the HBM theory lack of knowledge is related to poor understanding about the other constructs, including perceived susceptibility, perceived severity, and perceived barriers. These constructs, in part, affect self-efficacy and cue to action. Based on these observations and considering the HBM theory, it can be deduced that in studies that reported low level of knowledge in terms of breast cancer screening, the level of other HBM constructs should be low. This theory was proven in the reviewed studies. Previous studies have also indicated that when the level of knowledge of participants was low, their health behavior was also low and the interventions that affected the level of knowledge of participants regarding health behavior was improved ([56-58](#_ENREF_56)). However, the findings of previous studies about the effect of perceived severity and susceptibility and health behavior were controversial. Some studies showed that the relationship between perceived severity and susceptibility were not necessarily related to healthy behavior ([59](#_ENREF_59), [60](#_ENREF_60)), while other studies indicated that these constructs were related to health behavior ([61](#_ENREF_61), [62](#_ENREF_62)). The findings of the current review indicated that these constructs were related to breast cancer screening behavior. Therefore, it can be concluded that HBM theory can be used to determine the breast cancer screening behavior of Middle Eastern women. In order to improve the breast cancer screening behavior of Middle-Eastern women, interventions should focus on knowledge, and empowerment of the at risk population based on HBM theory.

To the best of our knowledge this systematic review was the first study that tried to synthesize the results of previous studies about predicting factors for breast cancer screening in Middle-Eastern women. One of limitations of this review was including studies that used different questionnaires for assessing the constructs of HBM regarding breast cancer prevention behavior. We suggest that the future studies focus on developing standard questionnaires regarding HBM constructs for preventing behaviors of breast cancer.

**Conclusion**

Considering the findings of this review, HBM can be used to predict breast cancer screening behavior of the Middle Eastern women. This theory can be used to improve breast cancer screening behavior. Interventions should consider all HBM constructs as the studied women had poor status in majority of these constructs. Regarding the crucial role of knowledge, it is recommended that education programs should be designed to improve all constructs of HBM model regarding breast cancer screening.

**Abbreviations**

BCE Breast Clinical examination

BSE Breast Self-Examination

HBM Health Belief Model

JBI Joanna Briggs Institute

MeSH Medical Subjects Heading

PRISMA Preferred Reporting Items for Systematic Reviews and Meta-Analyses

**References**

1. Zaidi Z, Dib HA. The worldwide female breast cancer incidence and survival, 2018. AACR; 2019.

2. Zhang M, Peng P, Wu C, Gong Y, Zhang S, Chen W, et al. Report of breast cancer incidence and mortality in China registry regions, 2008-2012. Zhonghua zhong liu za zhi [Chinese journal of oncology]. 2019;41(4):315-20.

3. DeSantis CE, Ma J, Gaudet MM, Newman LA, Miller KD, Goding Sauer A, et al. Breast cancer statistics, 2019. CA: a cancer journal for clinicians. 2019;69(6):438-51.

4. Azamjah N, Soltan-Zadeh Y, Zayeri F. Global Trend of Breast Cancer Mortality Rate: A 25-Year Study. Asian Pac J Cancer Prev. 2019;20(7):2015-20. PubMed PMID: 31350959. eng.

5. Berek, Noka's. Gynecology. 15, editor2012.

6. Rim SH, Allaire BT, Ekwueme DU, Miller JW, Subramanian S, Hall IJ, et al. Cost-effectiveness of breast cancer screening in the National Breast and Cervical Cancer Early Detection Program. Cancer Causes Control. 2019;30(8):819-26. PubMed PMID: 31098856. Epub 05/16. eng.

7. Kunst N, Long JB, Xu X, Busch SH, Kyanko KA, Richman IB, et al. Use and Costs of Breast Cancer Screening for Women in Their 40s in a US Population With Private Insurance. JAMA internal medicine. 2020 May 1;180(5):799-801. PubMed PMID: 32202606. Epub 2020/03/24. eng.

8. Nahidi F, Dolatian M, Roozbeh N, Asadi Z, Shakeri N. Effect of health-belief-model-based training on performance of women in breast self-examination. Electronic physician. 2017;9(6):4577.

9. Shojaeezadeh D. Study of behavioral models in health education. Tehran: Assistance of Ministry of Health. 2000:29-30.

10. Sharma M. Theoretical foundations of health education and health promotion. 2010.

11. Tussing L, Chapman-Novakofski K. Osteoporosis prevention education: behavior theories and calcium intake. Journal of the American Dietetic Association. 2005;105(1):92-7.

12. Tavafian S, Ramazanzadeh F. Socioeconomic characteristics and induced abortion: a cross sectional study based on health belief model. 2007.

13. Glanz K, Rimer BK, Viswanath K. Health behavior and health education: theory, research, and practice: John Wiley & Sons; 2008.

14. Mahmoud AA, Abosree TH, Abd El Aliem RS. Effect of The Health Belief Model-Based Education on Preventive Behaviors of Breast Cancer. Evidence-Based Nursing Research. 2020;2(4):11-.

15. Bal MD, Şahiner NC. The Effect of Health Belief Model Based Training on Cervical Cancer Screening Behaviors. Clinical and Experimental Health Sciences. 2020;10(3):223-7.

16. Rakhshanderou S, Maghsoudloo M, Safari-Moradabadi A, Ghaffari M. Theoretically designed interventions for colorectal cancer prevention: A Case of the Health Belief Model. 2020.

17. Kirag N, Kızılkaya M. Application of the Champion Health Belief Model to determine beliefs and behaviors of Turkish women academicians regarding breast cancer screening: A cross sectional descriptive study. BMC women's health. 2019;19(1):1-10.

18. Darvishpour A, Vajari SM, Noroozi S. Can health belief model predict breast cancer screening behaviors? Open access Macedonian journal of medical sciences. 2018;6(5):949.

19. Didarloo A, Nabilou B, Khalkhali HR. Psychosocial predictors of breast self-examination behavior among female students: an application of the health belief model using logistic regression. BMC public health. 2017;17(1):1-8.

20. Dewi TK, Massar K, Ruiter RA, Leonardi T. Determinants of breast self-examination practice among women in Surabaya, Indonesia: an application of the health belief model. BMC public health. 2019;19(1):1-8.

21. Ghaffari M, Esfahani SN, Rakhshanderou S, Koukamari PH. Evaluation of health belief model-based intervention on breast cancer screening behaviors among health volunteers. Journal of Cancer Education. 2019;34(5):904-12.

22. Khiyali Z, Aliyan F, Kashfi SH, Mansourian M, Jeihooni AK. Educational intervention on breast self-examination behavior in women referred to health centers: Application of Health Belief Model. Asian Pacific journal of cancer prevention: APJCP. 2017;18(10):2833.

23. Mahmoud MH, Sayed SH, Ibrahim HA-F, Abd-Elhakam EM. Effect of Health Belief Model-Based Educational Intervention About Breast Cancer on Nursing Students' Knowledge, Health Beliefs and Breast Self-Examination Practice. International Journal of Studies in Nursing. 2018;3(3):77.

24. Wang X, Chen D, Xie T, Zhang W. Predicting women's intentions to screen for breast cancer based on the health belief model and the theory of planned behavior. Journal of Obstetrics and Gynaecology Research. 2019;45(12):2440-51.

25. Becker MH, Maiman LA, Kirscht JP, Haefner DP, Drachman RH. The health belief model and prediction of dietary compliance: A field experiment. Journal of Health and Social behavior. 1977:348-66.

26. Harrison JA, Mullen PD, Green LW. A meta-analysis of studies of the health belief model with adults. Health education research. 1992;7(1):107-16.

27. Institute JB. The Joanna Briggs Institute. Joanna Briggs Institute Reviewers’ Manual: 2014 edition. The Joanna Briggs Institute. 2014.

28. Munn Z, Moola S, Lisy K, Riitano D, Tufanaru C. Methodological guidance for systematic reviews of observational epidemiological studies reporting prevalence and cumulative incidence data. International journal of evidence-based healthcare. 2015;13(3):147-53.

29. Taymoori P, Habibi S. Application of a health belief model for explaining mammography behavior by using structural equation model in women in Sanandaj. Scientific Journal of Kurdistan University of Medical Sciences. 2014;19(3):103-15.

30. Mokhtari L, Khorami Markani A, Habibpoor Z. Correlation between health beliefs and breast cancer early detection behaviors among females referring to health centers in Khoy city, Iran. Health Prom Manage. 2014;3(4):56-64.

31. Heydari E, Noroozi A, Tahmasebi R. The Impact of Education Based on Health Belief Model on Mammography among Bushehrian Teachers. Iranian Journal of Health Education and Health Promotion. 2017;4(4):271-80.

32. jadgal K, Zareban I, Faryabi R, Rafie M, AizadehSIUKI H. The Investigation of the Impact of Health Belief Model Based Training on Brest Self-Exam in Women Referred to Health Centers. Journal of Health Literacy. 2016;1(3):172-81. eng %@ 2476-471X %[ 2016.

33. Hajian-Tilaki K, Auladi S. Health belief model and practice of breast self-examination and breast cancer screening in Iranian women. Breast cancer. 2014;21(4):429-34.

34. Karayurt O, Dramal A. Adaptation of Champion's Health Belief Model Scale for Turkish women and evaluation of the selected variables associated with breast self-examination. Cancer Nursing. 2007;30(1):69-77.

35. MASOUDI YL, Dashtbozorgi B, Gheibizadeh M, SAKI MA, Moradi M. Applying the health belief model in predicting breast cancer screening behavior of women. 2015.

36. Sahraee A, Noroozi A, Tahmasebi R. Predicting Factors of Breast Self-Examination based on Health Belief Model and Locus of Control among Women Aged 20-50 Years. Hayat. 2013;19(2):27-39. eng %@ 1735-2215 %[ 2013.

37. Tahmasebi R, Hosseini F, Noroozi A. The effect of education based on the health belief model on women’s practice about Pap smear test. Hayat. 2016;21(4):80-92. eng.

38. Kasmaei p, bab eghbal s, Atrkare Roshan z, Estebsari f, Mehrabian f, Karimi m. THE EFFECT OF HEALTH BELIEF MODEL ON RURAL WOMEN'S PAP SMEAR TEST. Journal of Nursing and Midwifery Urmia University of Medical Sciences. 2014;12(5):401-8. eng.

39. Guilford K, McKinley E, Turner L. Breast Cancer Knowledge, Beliefs, and Screening Behaviors of College Women: Application of the Health Belief Model. American Journal of Health Education. 2017 2017/07/04;48(4):256-63.

40. karimy m, taher m, azarpira h. Measure health belief model construct about nutritional practices of pregnant women in Saveh University of Medical Sciences. Scientific Journal of Hamadan Nursing & Midwifery Faculty. 2016;24(3):167-73. eng.

41. Ramezankhani A, Jahani H, Hatami H, Sharifzadeh G, Hosseini S. Determine the effect of intervention on the adoption of preventive behaviours of the brucellosis was based on the health belief model. Journal of North Khorasan University of Medical Sciences. 2016;8(1):33-45. eng.

42. Fakhri A, Morshedi H, Zeidi I. Effect of education based on health belief model with relaxation on anxiety of nulliparouse women. Scientific Journal of Kurdistan University of Medical Sciences. 2017;22(2).

43. Dewi TK, Massar K, Ruiter RAC, Leonardi T. Determinants of breast self-examination practice among women in Surabaya, Indonesia: an application of the health belief model. BMC Public Health. 2019 2019/11/27;19(1):1581.

44. Taklual W, Tesfaw A, Mekie M, Shemelis T. Breast Self-Examination Practice among Female Undergraduate Students in Debre Tabor University, North Central Ethiopia: Based on Health Belief Model. Middle East Journal of Cancer. 2021.

45. Navabi M, Khorsandi M, Rouzbahani N, Ranjbaran M. The Effect of Education on Preventive Behaviors of Failure to Thrive in Mothers with Children Aged One to Five Years: Applied Health Belief Model. Arak Medical University Journal. 2017;20(1):59-68. eng.

46. Ziaee R, Jalili Z, Tavakoli Ghouchani H. The effect of education based on Health Belief Model (HBM) in improving nutritional behaviors of pregnant women. Journal of North Khorasan University of Medical Sciences. 2017;8(3):427-37. eng.

47. Tol A, Majlesi F, Shojaeizadeh D, Esmaelee Shahmirzadi S, Mahmoudi Majdabadi5 M, Moradian6 M. Effect of the educational intervention based on the health belief model on the continuation of breastfeeding behavior. 2 Journal of Nursing Education. 2013;2(2):39-47. eng.

48. Wang W-L, Hsu S-D, Wang J-H, Huang L-C, Hsu W-L. Survey of breast cancer mammography screening behaviors in Eastern Taiwan based on a health belief model. The Kaohsiung journal of medical sciences. 2014;30(8):422-7.

49. Bahri N, Mohebi S, Bahri N, Davoudi Farimani S, Khodadoost L. Factors related to the decision making process of primigravid women about mode of delivery: A theory-based study. The Iranian Journal of Obstetrics, Gynecology and Infertility. 2017;20(8):42-50.

50. Khorsandi M, Fekrizadeh Z, Roozbahani N. Investigation of the effect of education based on the health belief model on the adoption of hypertension-controlling behaviors in the elderly. Clinical interventions in aging. 2017;12:233-40. PubMed PMID: 28184154. Pubmed Central PMCID: PMC5291452. Epub 2017/02/12. eng.

51. Birhane N, Mamo A, Girma E, Asfaw S. Predictors of breast self-examination among female teachers in Ethiopia using health belief model. Archives of Public Health. 2015;73(1):39.

52. Marmarà D, Marmarà V, Hubbard G. Health beliefs, illness perceptions and determinants of breast screening uptake in Malta: a cross-sectional survey. BMC public health. 2017;17(1):416.

53. Babazadeh T, Nadrian H, Rezakhani Moghaddam H, Ezzati E, Sarkhosh R, Aghemiri S. Cognitive determinants of cervical cancer screening behavior among housewife women in Iran: An application of Health Belief Model. Health Care for Women International. 2018:1-16.

54. Namdar A, Bigizadeh S, Naghizadeh MM. Measuring Health Belief Model components in adopting preventive behaviors of cervical cancer. Journal of Fasa University of Medical Sciences. 2012;2(1):34-44. eng.

55. Doede AL, Mitchell EM, Wilson D, Panagides R, Oriá MOB. Knowledge, beliefs, and attitudes about breast Cancer screening in Latin America and the Caribbean: an in-depth narrative review. Journal of global oncology. 2018;4:1-25.

56. Simbar M, Ghazanfarpour M, Abdolahian S. Effects of training based on the health belief model on Iranian women's performance about cervical screening: A systematic review and meta-analysis. J Educ Health Promot. 2020;9:179-. PubMed PMID: 32953907. eng.

57. Victoria SA, Racquel E K, Lucila S, Melisa P, Viswanath K, Silvina A. Knowledge and perceptions regarding triage among human papillomavirus–tested women: A qualitative study of perspectives of low-income women in Argentina. Women's Health. 2020;16:1745506520976011.

58. Zare M, Ghodsbin F, Jahanbin I, Ariafar A, Keshavarzi S, Izadi T. The Effect of Health Belief Model-Based Education on Knowledge and Prostate Cancer Screening Behaviors: A Randomized Controlled Trial. Int J Community Based Nurs Midwifery. 2016;4(1):57-68. PubMed PMID: 26793731. eng.

59. Walrave M, Waeterloos C, Ponnet K. Adoption of a contact tracing app for containing COVID-19: A health belief model approach. JMIR public health and surveillance. 2020;6(3):e20572.

60. Karimy M, Bastami F, Sharifat R, Heydarabadi AB, Hatamzadeh N, Pakpour AH, et al. Factors related to preventive COVID-19 behaviors using health belief model among general population: a cross-sectional study in Iran. BMC Public Health. 2021 2021/10/24;21(1):1934.

61. Vogel EA, Henriksen L, Schleicher NC, Prochaska JJ. Perceived Susceptibility to and Seriousness of COVID-19: Associations of Risk Perceptions with Changes in Smoking Behavior. International Journal of Environmental Research and Public Health. 2021;18(14):7621.

62. Keren F, Siddiquei AN, Anwar MA, Asmi F, Ye Q. What Explains Natives and Sojourners Preventive Health Behavior in a Pandemic: Role of Media and Scientific Self-Efficacy. Frontiers in Psychology. 2021 2021-June-29;12. English.

**Table 1. Level of the reported constructs of HBM in the reviewed studies**

| Author (Year) | **Study characteristics** | | | **Health belief model constructs** | | | | | | | | |
| --- | --- | --- | --- | --- | --- | --- | --- | --- | --- | --- | --- | --- |
|  | Setting | N | Study population | Knowledge | Perceived susceptibility | Perceived severity | Perceived benefits | Perceived barriers | Cues to action | Action plan | Self-efficacy | behavior |
| Taymoori (2014) | Sanandaj, Iran | 593 | Clusters of women referring to health centers (>40 years old) | - | Poor | Poor | Poor | Poor | Poor | - | Poor | - |
| Mokhtari (2014) | Khoy, Iran | 162 | Clusters of women referring to health centers (mean age 29 years old) | - | Moderate | - | Good | Good | Good | Moderate | - | Moderate |
| Heydari (2017) | Bushehr, Iran | 400 | Teachers (>40 years old) | - | Poor | Poor | Poor | Poor | Poor | - | Poor | - |
| Jadgal (2016) | Zahedan, Iran | 240 | Clusters of women referring to health centers (mean age 28 years old | - | Good | Good | Good | Good | Good | - | - | Moderate |
| Hajian-Tilaki (2012) | Babol, Iran | 500 | Clusters of women referring to health centers (18-65 years old | Poor | Poor | Poor | Poor | Good | Good | Poor | Good | Poor |
| Karayurt (2007) | Izmir, Turkey | 430 | Women referring to health centers (20-60 years old | - | Good | Good | - | Good | Good | - | Good | - |
| Masoudi (2015) | Dezful, Iran | 226 | Clusters of women referring to health centers (20-60 years old | Poor | Poor | Poor | Poor | Poor | - | Poor | Poor | - |
| Shahraee (2013) | Bushehr, Iran | 400 | Women referring to health centers (20-50 years old | - | Moderate | Poor | Poor | Moderate | - | - | Moderate | poor |


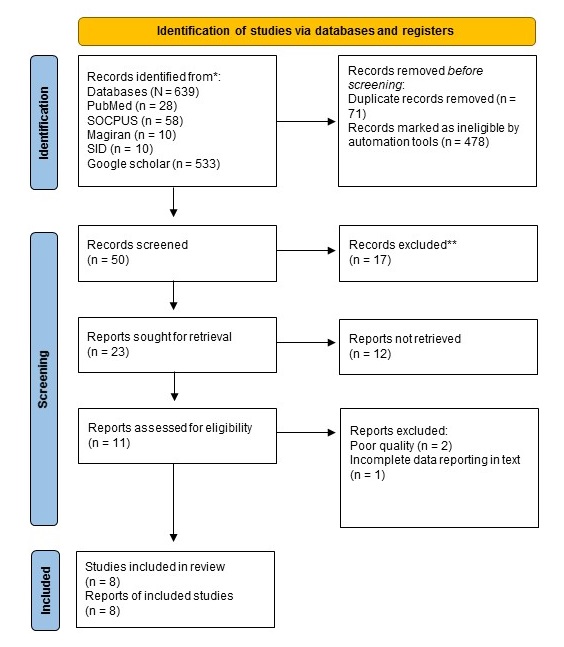


**Figure 1. Successive steps in the selection of studies—PRISMA Flow Diagram**

**Supplementary files 1. Search strategy for the systematic review**

| Search term | Search bibliography |
| --- | --- |
| Breast cancer | Breast neoplasm  Breast cancer |
| Mammography | Mammography  Mammogram |
| Brest self-examination | Breast self-examination  Breast self-examination  Breast self-exam |
| Health belief model | Health belief model  HBM |
| Middle-East | Middle east  Middle eastern |

PubMed Search query

| Database | Search strategy |
| --- | --- |
| PubMed | # 1. MeSH descriptor Breast Neoplasms explode all trees  # 2. (breast):ti  # 3. (#1 OR #2)  # 4. MeSH descriptor Neoplasm Recurrence, Local explode all trees  # 5. #3 OR #4  #6. MeSH descriptor Mammography explode all trees  # 7. (mammograph* or mammogram*):ti,ab,kw  # 8. MeSH descriptor Physical Examination, this term only  # 9. MeSH descriptor Breast Self-Examination, this term only  # 10. (#6 OR #7 OR #8 OR #9)  # 11. Middle East  #12. MiddleEast  #13. (#11 OR #12)  #14. (#3 AND #5 AND #9 AND #13) |
| SCOPUS | #1. TITLE-ABS-KEY ( "breast neoplasm" OR "breast cancer" OR "breast carcinoma" ) AND DOCTYPE ( ar )  #2. TITLE-ABS-KEY ("mammography" OR "mammogram" ) AND DOCTYPE ( ar )  #3. TITLE-ABS-KEY (screening) AND DOCTYPE ( ar )  #4. #2 OR #3  #5. TITLE-ABS-KEY ("breast" AND ("physical examination" OR "self examination" OR "self-examination") AND DOCTYPE ( ar )  #6. #1 AND #3 AND #5 |

**Supplementary files-2. PRISMA checklist of the review**

| **Section and Topic** | **Item #** | **Checklist item** | **Location where item is reported** |
| --- | --- | --- | --- |
| **TITLE** | | |  |
| Title | 1 | Identify the report as a systematic review. | Title, abstract and methods |
| **ABSTRACT** | | |  |
| Abstract | 2 | See the PRISMA 2020 for Abstracts checklist. | Abstract |
| **INTRODUCTION** | | |  |
| Rationale | 3 | Describe the rationale for the review in the context of existing knowledge. | Introduction |
| Objectives | 4 | Provide an explicit statement of the objective(s) or question(s) the review addresses. | Introduction (last paragraph) |
| **METHODS** | | |  |
| Eligibility criteria | 5 | Specify the inclusion and exclusion criteria for the review and how studies were grouped for the syntheses. | Methods (search strategy subheading) |
| Information sources | 6 | Specify all databases, registers, websites, organisations, reference lists and other sources searched or consulted to identify studies. Specify the date when each source was last searched or consulted. | Methods (search strategy) |
| Search strategy | 7 | Present the full search strategies for all databases, registers and websites, including any filters and limits used. | Methods (search strategy), Supplementary file 1 |
| Selection process | 8 | Specify the methods used to decide whether a study met the inclusion criteria of the review, including how many reviewers screened each record and each report retrieved, whether they worked independently, and if applicable, details of automation tools used in the process. | Methods (search strategy) |
| Data collection process | 9 | Specify the methods used to collect data from reports, including how many reviewers collected data from each report, whether they worked independently, any processes for obtaining or confirming data from study investigators, and if applicable, details of automation tools used in the process. | Figure 1. |
| Data items | 10a | List and define all outcomes for which data were sought. Specify whether all results that were compatible with each outcome domain in each study were sought (e.g. for all measures, time points, analyses), and if not, the methods used to decide which results to collect. | Methods (search strategy) |
|  | 10b | List and define all other variables for which data were sought (e.g. participant and intervention characteristics, funding sources). Describe any assumptions made about any missing or unclear information. | Not applicable |
| Study risk of bias assessment | 11 | Specify the methods used to assess risk of bias in the included studies, including details of the tool(s) used, how many reviewers assessed each study and whether they worked independently, and if applicable, details of automation tools used in the process. | Methods (Risk of Bias Assessment for Individual Studies subheading) |
| Effect measures | 12 | Specify for each outcome the effect measure(s) (e.g. risk ratio, mean difference) used in the synthesis or presentation of results. | Not applicable |
| Synthesis methods | 13a | Describe the processes used to decide which studies were eligible for each synthesis (e.g. tabulating the study intervention characteristics and comparing against the planned groups for each synthesis (item #5)). | Methods (search strategy) |
|  | 13b | Describe any methods required to prepare the data for presentation or synthesis, such as handling of missing summary statistics, or data conversions. | Not applicable |
|  | 13c | Describe any methods used to tabulate or visually display results of individual studies and syntheses. | Results |
|  | 13d | Describe any methods used to synthesize results and provide a rationale for the choice(s). If meta-analysis was performed, describe the model(s), method(s) to identify the presence and extent of statistical heterogeneity, and software package(s) used. | Not applicable |
|  | 13e | Describe any methods used to explore possible causes of heterogeneity among study results (e.g. subgroup analysis, meta-regression). | Not applicable |
|  | 13f | Describe any sensitivity analyses conducted to assess robustness of the synthesized results. | Not applicable |
| Reporting bias assessment | 14 | Describe any methods used to assess risk of bias due to missing results in a synthesis (arising from reporting biases). | Not applicable |
| Certainty assessment | 15 | Describe any methods used to assess certainty (or confidence) in the body of evidence for an outcome. | Not applicable |
| **RESULTS** | | |  |
| Study selection | 16a | Describe the results of the search and selection process, from the number of records identified in the search to the number of studies included in the review, ideally using a flow diagram. | Results and Figure 1 |
|  | 16b | Cite studies that might appear to meet the inclusion criteria, but which were excluded, and explain why they were excluded. | Figure 1 |
| Study characteristics | 17 | Cite each included study and present its characteristics. | Results, Table 1 |
| Risk of bias in studies | 18 | Present assessments of risk of bias for each included study. | Not applicable |
| Results of individual studies | 19 | For all outcomes, present, for each study: (a) summary statistics for each group (where appropriate) and (b) an effect estimate and its precision (e.g. confidence/credible interval), ideally using structured tables or plots. | Results and Table 1 |
| Results of syntheses | 20a | For each synthesis, briefly summarise the characteristics and risk of bias among contributing studies. | Table 1 |
|  | 20b | Present results of all statistical syntheses conducted. If meta-analysis was done, present for each the summary estimate and its precision (e.g. confidence/credible interval) and measures of statistical heterogeneity. If comparing groups, describe the direction of the effect. | Not applicable |
|  | 20c | Present results of all investigations of possible causes of heterogeneity among study results. | Not applicable |
|  | 20d | Present results of all sensitivity analyses conducted to assess the robustness of the synthesized results. | Not applicable |
| Reporting biases | 21 | Present assessments of risk of bias due to missing results (arising from reporting biases) for each synthesis assessed. | Not applicable |
| Certainty of evidence | 22 | Present assessments of certainty (or confidence) in the body of evidence for each outcome assessed. | Not applicable |
| **DISCUSSION** | | |  |
| Discussion | 23a | Provide a general interpretation of the results in the context of other evidence. | Discussion and conclusion |
|  | 23b | Discuss any limitations of the evidence included in the review. | Discussion |
|  | 23c | Discuss any limitations of the review processes used. | Discussion |
|  | 23d | Discuss implications of the results for practice, policy, and future research. | Discussion and conclusion |
| **OTHER INFORMATION** | | |  |
| Registration and protocol | 24a | Provide registration information for the review, including register name and registration number, or state that the review was not registered. | Not applicable |
|  | 24b | Indicate where the review protocol can be accessed, or state that a protocol was not prepared. | Supplementary file 1 |
|  | 24c | Describe and explain any amendments to information provided at registration or in the protocol. | Not applicable |
| Support | 25 | Describe sources of financial or non-financial support for the review, and the role of the funders or sponsors in the review. | Not applicable |
| Competing interests | 26 | Declare any competing interests of review authors. | Acknowledgment |
| Availability of data, code and other materials | 27 | Report which of the following are publicly available and where they can be found: template data collection forms; data extracted from included studies; data used for all analyses; analytic code; any other materials used in the review. | Not applicable |
